# Rare Presentation of Metastatic Lobular Breast Carcinoma Involving Clear Cell Renal Cell Carcinoma

**DOI:** 10.1155/2020/5315178

**Published:** 2020-09-14

**Authors:** Dayne Ashman, Gabriela Quiroga-Garza, Daniel Lee

**Affiliations:** ^1^Department of Pathology, University of Pittsburgh, 5230 Centre Avenue, Pittsburgh, PA 15232, USA; ^2^Department of Medicine, University of Pittsburgh, 5150 Centre Avenue, Pittsburgh, PA 15232, USA

## Abstract

Although the first case of tumor-to-tumor metastasis was reported over a century ago, it remains a rare occurrence. We report a rare case of metastatic infiltrating lobular carcinoma involving clear cell renal cell carcinoma, as well as offer a brief literature review.

## 1. Introduction

The phenomenon of one malignant tumor metastasizing to another, unrelated, primary tumor, has been termed “tumor-to-tumor” metastasis (TTM); it is a rare occurrence. To date, there have been fewer than 200 cases reported in the English literature. Here, we report a rare case of metastatic infiltrating lobular carcinoma involving clear cell renal cell carcinoma.

## 2. Case Report

**Figure 1:**
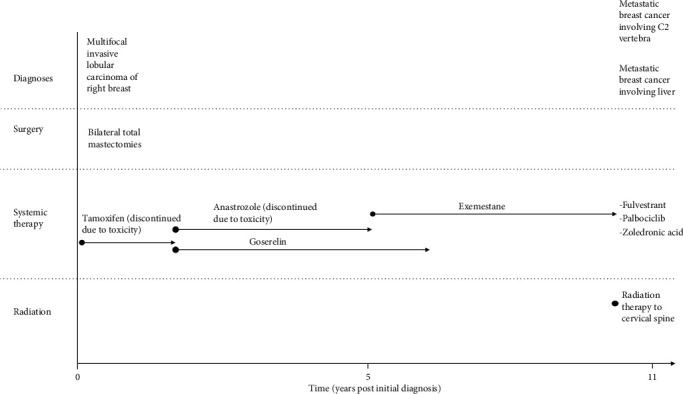
Overview of clinical history and management.

**Figure 2:**
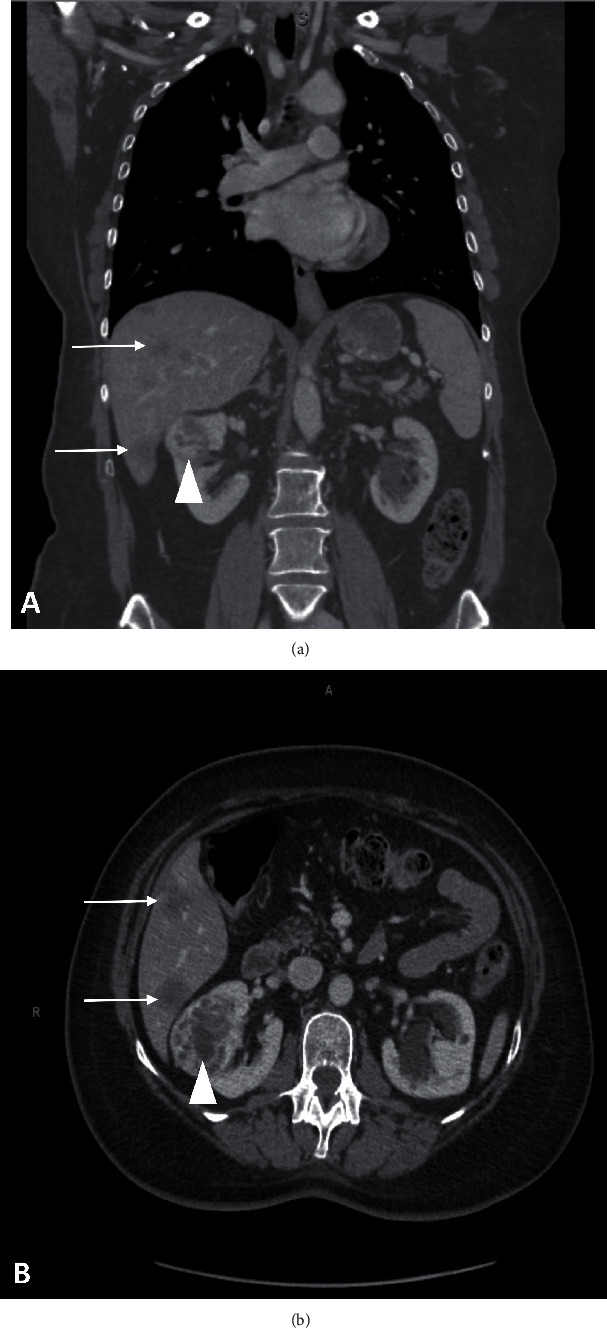
Abdominal CT scan with right renal mass (white arrowheads) and liver metastases (white arrows). (a) Coronal section. (b) Transverse section.

A 57-year-old woman with a past medical history of stage IIA (pT2 N0 M0) multifocal infiltrating lobular carcinoma of the right breast, and multifocal lobular carcinoma *in situ* of the left breast (both diagnosed 11 years prior), was initially managed with surgery and adjuvant hormonal therapy. An overview of her clinical history and management is given in Figure 1. She recently presented with a right renal mass, incidentally identified during computerized tomography (CT) scan performed as part of her follow-up (Figure 2). An ultrasound-guided needle biopsy of the right renal mass was performed.

**Figure 3:**
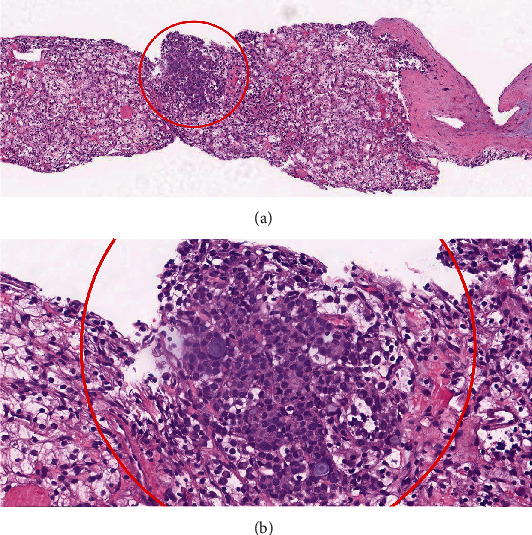
Metastatic infiltrating lobular breast carcinoma involving ccRCC. (a) At 40X, the encircled metastatic breast carcinoma focus is surrounded by ccRCC. (b) At 200X, intracytoplasmic mucin is apparent.

Microscopic examination of the biopsy specimen revealed two distinct neoplasms (Figure 3). The first neoplasm identified demonstrated cells arranged in nests and solid sheets with delicate vasculature. Cytologically, these neoplastic cells demonstrated clear cytoplasm with round nuclei and inconspicuous nucleoli, morphologically consistent with a clear cell renal cell carcinoma (ccRCC).

The second neoplasm was identified within the substance of the ccRCC and consisted of a fairly well-circumscribed 0.5 mm solid nest of cells, with a vaguely nodular architecture. Cytologically, this second population of neoplastic cells showed basophilic cytoplasm, with hyperchromatic nuclei exhibiting moderate nuclear pleomorphism. Some areas exhibiting intracytoplasmic mucin were also identified. No host reaction was apparent.

**Figure 4:**
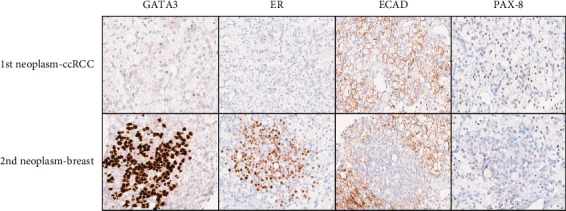
A panel of immunohistochemical stains performed supports the morphologic impression of two distinct neoplasms—ccRCC and metastatic lobular breast carcinoma. The neoplastic cells of the ccRCC are negative for GATA3 and ER, but immunoreactive for ECAD and PAX-8. The neoplastic cells of the metastatic lobular breast carcinoma are immunoreactive for GATA3 and ER, but negative for ECAD and PAX-8. Immunostains for CK7, CD10, and RCC antigen are not shown.

A panel of immunohistochemical stains was performed in order to confirm the identity of the two distinct neoplasms. The stains performed included: cytokeratin 7 (CK7), GATA3, estrogen receptor (ER), e-cadherin (ECAD), PAX-8, CD10, and renal cell carcinoma antigen (RCC) (see Figure 4). The morphologic impression and immunophenotype of the 1^st^ neoplasm were consistent with ccRCC. The morphologic impression and immunophenotype of the 2^nd^ neoplasm were consistent with metastatic lobular carcinoma of the breast.

The patient remains on fulvestrant and palbociclib with stable disease on the most recent scan. As renal lesions often grow slowly and have good prognosis, her scans will be closely monitored for a potential nephrectomy.

## 3. Discussion

Metastasis from one neoplasm (the donor) to another neoplasm (the recipient) was first reported over a century ago by Berent in 1902. This sentinel case involved squamous cell carcinoma of the jaw and renal cancer, as the donor and recipient, respectively. During the decades that followed, various authors reported this phenomenon, with the donor neoplasm arising from a variety of sites, including breast [1–3], lung [4–6], and less common sites such as skin [7] and prostate [8]. Frequently reported recipient tumors include renal cell carcinoma, meningioma, and sarcoma [9, 10], with renal neoplasms being the most common tumor metastasis recipient. To date, a variety of renal neoplasms have been reported to be involved in TTM as the recipient neoplasm, including renal oncocytoma [11, 12] as well as chromophobe RCC [13, 14], but ccRCC has been the most frequently reported recipient renal neoplasm. We report a case of metastatic invasive lobular carcinoma involving ccRCC. A few cases have been previously reported of metastatic breast cancer involving ccRCC, but most of these cases involved metastatic infiltrating ductal carcinoma [15, 16].

Although the exact mechanism by which tumor-to-tumor metastasis occurs is yet to be detailed, authors have relied on various theories to help explain this phenomenon, including the metabolic theory [17, 18] and the mechanical/anatomic theory [19]. The metabolic theory, also referred to as “the seed and soil theory”, was proposed by Stephen Paget in 1889 after carefully analyzing postmortem data of 735 patients with metastatic breast cancer. He concluded that metastatic tumor cells (the seed) would preferentially grow in microenvironments (the soil) with abundant micronutrients. In the years that followed, this hypothesis was challenged by some, including James Ewing, who proposed that tumor metastasis was mainly determined by hemodynamic factors of the vascular and lymphatic system, as these factors were most important for successful delivery of metastatic tumor cells [19].

Considering these two theories, it is understandable why renal cell carcinoma, and in particular-ccRCC, is such a frequent recipient tumor. The kidneys receive approximately 20% of the cardiac output (approximately 1.0 liter/minute) [20]. In ccRCC, von Hippel Lindau tumor suppressor gene is inactivated, which increases hypoxia-inducible factor, which in turn increases vascular endothelial growth factor. As a result, ccRCC is highly vascularized [21]. From a mechanistic perspective, this situation is hemodynamically favorable. From a metabolic perspective, ccRCC has increased glycogen and lipid content [22], which may account for it being a favorable microenvironment. With this in mind, it is easy to understand why, even decades ago, renal cell carcinoma was proclaimed to be the best recipient of tumor-to-tumor metastasis [10].
